# Assessment of diagnostic accuracy of confocal image features used in detection of hypopigmented verruca plana on children's forehead and classic verruca plana in adult

**DOI:** 10.1111/srt.13148

**Published:** 2022-04-13

**Authors:** An‐Kang Gu, Jing Shi, Xiu‐Jun Zhang, Fa‐Ku Ma, Yu Zhang

**Affiliations:** ^1^ Department of Pathology Tianjin Academy of Traditional Chinese Medicine Affiliated Hospital Tianjin China; ^2^ Department of Dermatology Tianjin Academy of Traditional Chinese Medicine Affiliated Hospital Tianjin China

## 1 INTRODUCTION

In clinical work, we found that some children's verruca plana (VP) lesions on the forehead are characterized by hypopigmented papules. In contrast to classic VP, hypopigmented VP is characterized by pale or milky papules, mostly occurs on children's foreheads, and is rarely seen in adults. Although the typical reflectance confocal microscopy (RCM) features of classic VP carried out by Liu and Hui are concentric circle structure,^1^, ^2^ which is like a cluster of roses, there have been few studies on RCM findings of hypopigmented VP. In the study of Gu and Chen,^3^, ^4^ they found hypopigmented VP showing large polygonal cells in the upper spinous layers or granular layers, which were three–five times as large as normal keratinocytes. Studies have confirmed that the confocal image features of large polygonal cells and concentric circle‐like structures with certain diagnostic value for VP^3^; however, to date, existing knowledge has not systematically evaluated the specificity and sensitivity of confocal image feature in detection of hypopigmented and classic VP. We conducted assessment of diagnostic accuracy of confocal image features used in detection of hypopigmented VP on children's forehead and classic VP in adult.

## 2 CASE SERIES AND METHODS

**FIGURE 1:**
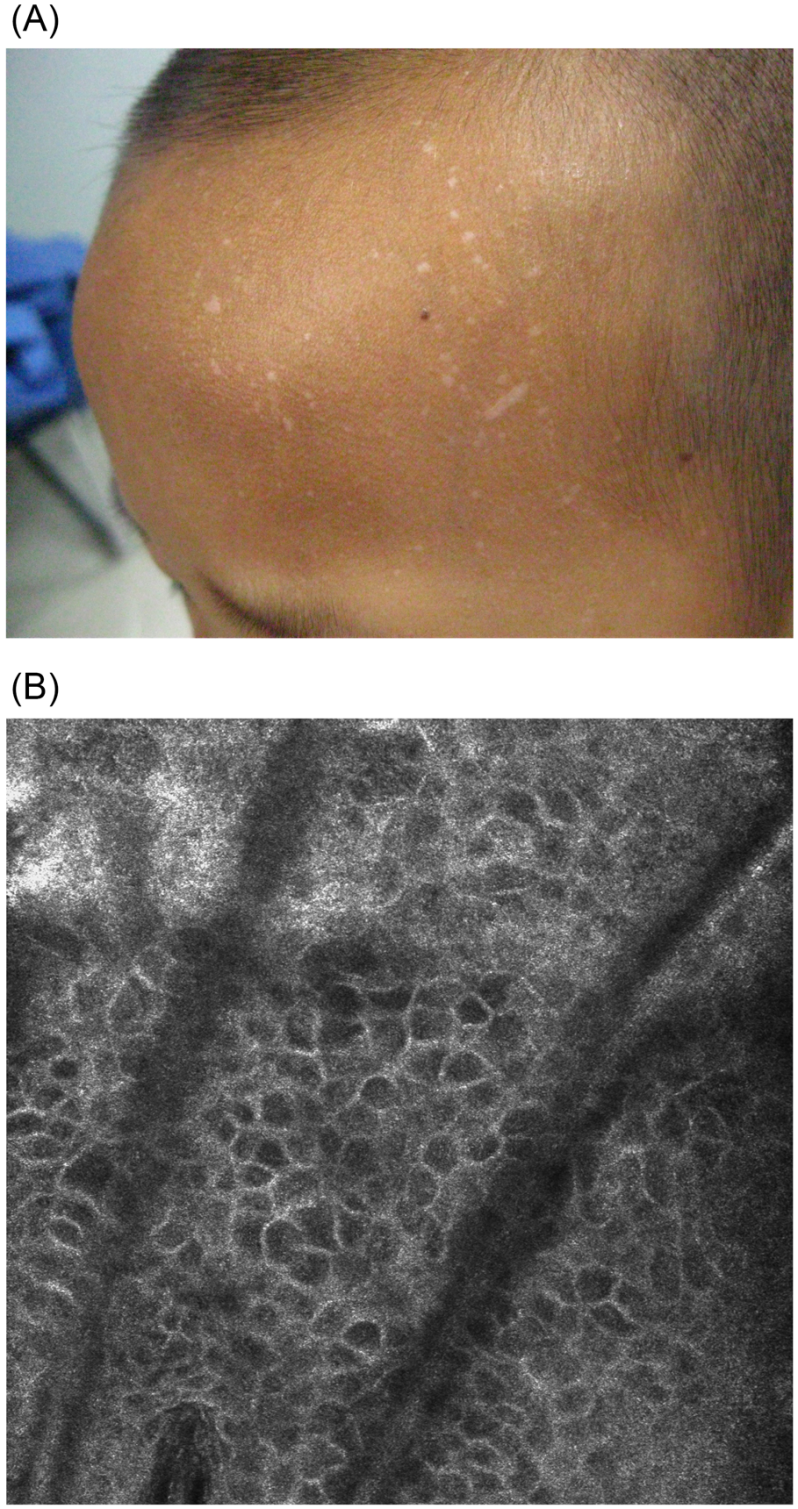
(A) Hypopigmented flat papules on a child's forehead. (B) Reflectance confocal microscopy (RCM) images showed large polygonal cells in the upper part of the spinous layer (depth 12 µm, level 0.5 mm × 0.5 mm)

**FIGURE 2:**
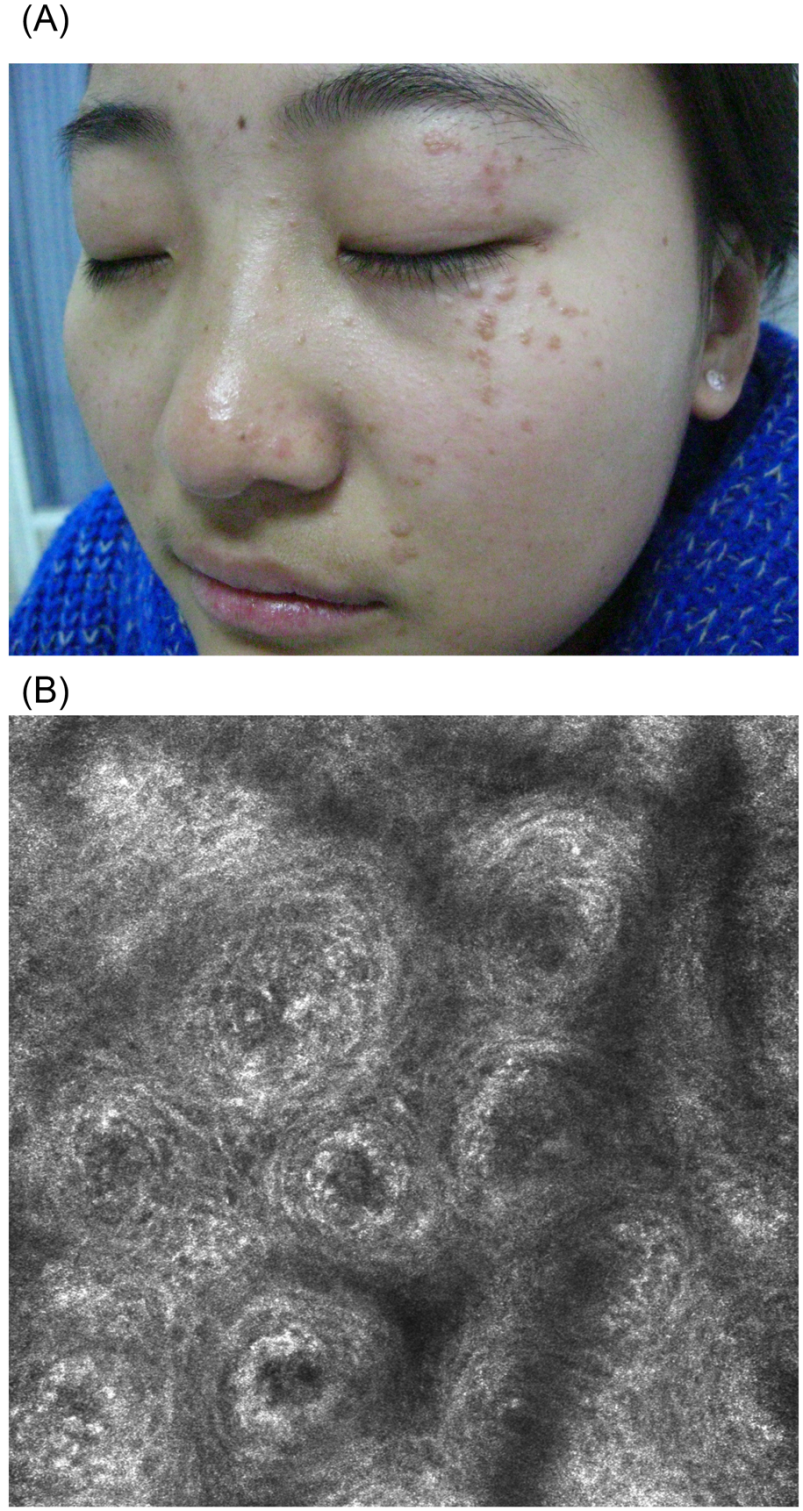
(A) Pigmented flat papules on an adult's face. (B) Typical concentric circle‐like structure in the upper spinous layer by reflectance confocal microscopy (RCM) (depth 19 µm, level 0.5 mm × 0.5 mm)

A total of 40 patients, 20 with hypopigmented lesions and 20 with classic lesions, were enrolled in this study. Twenty children (nine males and 11 females) with a clinical suspicious diagnosis of hypopigmented VP on the forehead between 2 and 11 years of age (mean age 6.06) had their disease course ranging from 1 to 14 months. The skin lesions were all white or milky papules of 2–5 mm in diameter, with smooth surface. Three cases showed fusion of the lesions, and four cases had pruritus with isomorphic reactions (Figure 1A). Twenty adults (eight males and 12 females) with classic VP on the face between 15 and 28 years of age (mean age 22.58) were selected. The course of the disease ranged from 5 months to 2.5 years. The skin lesions were all manifested as flesh or brown flat papules of 2–5 mm in diameter, with smooth surface and varying degrees of itching (Figure 2A). All these cases were recruited prospectively from Dermatology Department of Academy of Traditional Chinese Medicine Affiliated Hospital in Tianjin between June 2016 and January 2021.

All included cases were imaged with a commercially available, near infrared, reflectance mode confocal microscope (Vivascope 1500; Lucid Inc., Rochester, NY, USA), which used an 830‐nm laser beam with maximum power of 22 mW. Each image with a resolution of 1000 × 1000 pixels, corresponding to a 0.5 × 0.5 mm horizontal cross section, was stored using a Bitmap file format. After the RCM imaging, an excisional biopsy was performed on all included cases. Specimens were routinely fixed in phosphate‐buffered neutral formalin, embedded in paraffin, and sectioned vertically. Finally, 4‐µm thick sections were stained using hematoxylin and eosin (stain).

## 3 RESULTS

In 20 hypopigmented cases, histological findings revealed the presence of keratosis, slight acanthosis, and vacuolized keratinocytes in the granular layers or upper spinous layers. The pathological changes of 20 classic cases were similar to those in the study group with less vacuole cells and more prominent papillomatosis.

In 18 of 20 hypopigmented case, large polygonal cells were found in the upper spinous layers or granular layers by RCM, which were three–five times as large as normal keratinocytes (Figure 1B), and the rest two had no obvious specific changes. In six of the 18 (6/18; 33.3%), large polygonal cells were overlapping and/or transitioning with concentric circles in the upper part of the spinous layers, while typical concentric circles (rosette‐like changes) were seen in the middle part of epidermis. The course of these six kids (3, 6, 10, 12, 13, and 15 months) was longer than that of the remaining 14 children (all in 1–4 months).

In the classic group, 11 of 20 patients showed the main features of petal‐like structures in the stratum granulosum and the stratum spinosum by RCM (Figure 2B). However, 10 patients of these 11 cases (10/11; 90.91%) had the disease for more than 1 year. However large polygonal cells were detected in three female patients (6–10 months of medical history) without typical concentric structure, and the rest six had no obvious specific changes.

So large polygonal cells are the main RCM characteristic of hypopigmented VP. Its sensitivity is 90.0% (18/20), and specificity is 85.0% (17/20). Its diagnostic accuracy is 87.5% (35/40). The authors believe that the petal‐like structure has sensitivity of 55.0% (11/20) for classical VP and specificity of 70.0% (14/20). Its diagnostic accuracy is 62.5% (25/40).

## 4 DISCUSSION

RCM technology is a cost‐effective tool in the diagnosis of VP.^5^, ^6^ Its use has been associated with increases in the diagnosis accuracy for VP and decreases in the number of unnecessary skin biopsies. In our study, we affirmed the diagnostic importance of RCM structures associated with VP detection.

A total of 40 patients, including 20 hypopigmented cases and 20 classic VPs, were included to assess the diagnostic value of RCM structures used in VP detection. Hui et al^2^ performed dermoscopy and RCM tests on 55 adult pigmented VPs and pointed out that the main features of petal‐like structures had highly specific for VP without treatment. However, in their study, the petal‐like structures probability by RCM was not very high, only 49.1% and 88.89% of patients had the disease for more than 1 year. Therefore, they believed that the concentric circle‐like structure is a typical manifestation of mature VP, and it is difficult for RCM to identify the early stage of VP. However, in our study, VP with hypopigmented papules on children's forehead with a relatively short history showed large polygonal cells in the upper part of epidermis. Its sensitivity is 90.0% and specificity is 85.0%, with diagnostic accuracy of 87.5%.Only a small proportion of these children (33.3%) with a relatively long history may observe the typical concentric circles, and overlapping and/or transitioning areas of the two are visible, verifying that large polygonal cells are evidence of early infection and the typical concentric circles are characteristic of mature skin lesions.

In the classic group, 11 cases were observed typical concentric circle structures without large polygonal cells, 10 of whom had the disease for more than 1 year. Its sensitivity is 55% and specificity is 70.0%, with diagnostic accuracy of 62.5%. This result was close to that of Hui’s team^2^ but lower than Lu's team with sensitivity of 78.07%.^7^ Large polygonal cells were also detected in three female patients of classic VPs with a relatively short medical history, proving that this structure is a characteristic of early lesions of flat warts from the other side. As the lesions mature, the epidermis thickens, and papilloma structures developed, typical concentric structures can be detected by RCM. However, in actual clinical practice, we see the similar petal‐like structures in skins of hands or feet under RCM. However, this is usually not a problem for experienced dermatologists because the clinical presentation and location of the disease are sufficient to identify its diagnostic value.

## 5 CONCLUSION

We believe that the petal‐like structure is highly specific for mature VP and that the large polygonal cell is the main characteristic of hypopigmented VP or early lesions. As expected, the large polygonal structures associated with hypopigmented VP detection tended to have higher specificity and sensitivity compared with classical VP with petal‐like structure. Therefore, noninvasive imaging techniques, such as RCM, have high diagnostic value for VP, especially hypopigmented type.

## CONFLICT OF INTEREST

The authors declare that there is no conflict of interest that could be perceived as prejudicing the impartiality of the research reported.
